# Factors associated with contracted services of Chinese family doctors from the perspective of medical staff and consumers: a cross-sectional study

**DOI:** 10.1186/s12913-019-4801-y

**Published:** 2019-12-21

**Authors:** Huanyan Wang, Lei Shi, Xuanye Han, Jinchan Zhang, Yuanshuo Ma, Xi Yang, Ming Liu, Lihua Fan, Fengge Lou

**Affiliations:** 1 Department of Health Management, School of Health Management, Harbin Medical University, No.157 Baojian Road, Nangang District, Harbin, 150081 China; 2 Human Resource Office, Women’s Hospital School of Medicine, Zhejiang University, Hangzhou, Zhejiang China; 3 Department of Neurosurgery, The Second Affiliated Hospital of Harbin Medical University, Harbin, Heilongjiang China; 4 Department of Medical Dispute, Maternal and Child Health Hospital, Heyuan, Guangdong China; 5 Department of Public Health Research, School of Public Health, Qiqihar Medical University, Qiqihar, Heilongjiang China

**Keywords:** Family doctors, Related factors, Contracted services, Patients

## Abstract

**Background:**

The family doctor system has developed rapidly all over the world, and in the past few years, China has actively explored family doctor-type contracted services. This study aimed to explore the related factors of Contracted Family Doctors Services (CFDS) from the perspectives of medical staff and consumers, and to provide a stronger basis for the development and promotion of CFDS.

**Methods:**

A combination of quantitative and qualitative methods were used in this study. A self-reported questionnaire was designed through a literature analysis, group discussions, expert consultations and a pre-investigation, and conducted among community health service providers in 12 community health service centres across four provinces of China. A total of 389 participants participated, and 320 valid questionnaires were obtained, with an effective response rate of 82.3%. A total of 36 consumers participated in in-depth interviews, and the effective rate was 100.0%. An exploratory factor analysis, a confirmatory factor analysis, inductive methods, and expert consultations were used to analyse the related factors of CFDS.

**Results:**

The related factors of CFDS from the perspectives of medical staff were divided into four dimensions, with the following weighting coefficients: national government factors (31.9%), community health service agency factors (24.7%), consumer-related factors (22.6%), and contracted doctor-related factors (20.8%). The related factors of CFDS from the perspectives of consumers were divided into four dimensions, with the following frequency sequence: national government factors, contract doctor-related factors, community health service agency factors, and consumers-related factors. National government factors played an important role in CFDS from the perspectives of medical staff and consumers.

**Conclusions:**

The related factors of CFDS were the same from the perspective of medical staff and consumers, but the weight of each factor was different. The development of CFDS is inseparable from the support of policies. It is suggested that the government should strengthen the publicity of CFDS, expand the coverage, introduce personalised contract programs that meet the needs of different groups, and promote the rapid development of CFDS.

## Background

The family doctor system, as an important policy measure to realize the *Alma-Ata Declaration’s* grand goal of primary health care for all, has gained rapid ground worldwide [1]. According to a World Health Organization report, the proportion of all patients with diseases requiring specialized medical treatment was only about 5%, and more than 90% of health problems could be effectively solved by professionally trained general practitioners [2]. The role of general practitioners/family doctors, according to the World Organization of Family Doctors, is to provide comprehensive health care services and to arrange for other health professionals to provide related services when necessary [3].

The family doctor system has been implemented in more than 50 countries and regions and has achieved gratifying results in various respects, which has attracted the attention of governments and medical circles [4]. There exist obvious differences in the specific service modes and operating mechanisms in various countries. Moreover, there is no doubt that the family doctor system plays an important role in medical and health service systems. The family doctor system originated in the United States in the 1960s [5], when the government integrated health management into the community general practitioner service model [6]. At the same time, active follow-up observations were performed on patients with chronic diseases and during the rehabilitation period [6].

In the U.K., the National Health Service was established in 1948 and adopted the national management model, which requires citizens to register with their family doctors and sign contracts with them [7]. Cuba established the family doctor program, in which a family physician and at least one nurse were each responsible for providing primary disease prevention and medical treatment services to 120–150 families [8]. The family doctor system in Denmark is a form of health care developed from the private doctor system, and each family doctor is responsible for a maximum of 1780 health care-registered patients /consumers [9].

However, in China, the idea of ‘family doctors’ was introduced in the 1980s, when ‘family doctor-type contracted services’ were proposed. In 2003, the Chinese government put forward the family doctor system as the goal of the development of community health services so that everyone can benefit from family doctor services [10]. The promulgation of the Guiding Opinions of the State Council on the Establishment of the General Practitioner System in 2011 indicates that the establishment of the family doctor system has become national strategy [11]. Subsequently, the training objectives, methods, and contents, and the ability requirements of general practitioners were further clarified [12]. *The Guideline on Promoting Contracted Family Doctor Services* was issued to accelerate family doctor contract services [12]. To improve quality and efficiency the National Health and Family Planning Commission also pointed out that the work of family doctors has changed [13], and the contracted family doctor services (CFDS) have been promoted in a systematic way across China.

In recent years, China has been exploring the implementation of family doctor-type contracted services. At present, five typical contracted service models have been formed; for example, the ‘1 + 1 + 1’ contracted service model in Shanghai [14], ‘basic package + personalized package’ contracted service model in Yancheng, Jiangsu Province [15], ‘integrated Medical treatment and nursing care system’ contracted service model in Hangzhou, Zhejiang Province [16], ‘co-management of doctors of three kinds’ contracted service model in Xiamen, Fujian Province [17], and ‘capitation prepayment’ contracted service model in Dingyuan, Anhui Province [15].

The research on family doctors in China has mainly focused on the significance and difficulties of establishing a family doctors system, or it has explored the policy effects of family doctors in terms of service utilization, management of non-communicable diseases, medical expenses, and satisfaction [18–21]. Some researchers have also noticed the importance of establishing stable relationships with family doctors, as well as contract status and contract behaviour [22, 23]. Wenjuan and colleagues studied the primary health service capacity in Western China from different doctor and patient perspectives, and found that coordination is the weakest component of the primary health service ability [24]. A cross-sectional study indicated that the awareness rate of residents on CFDS is 71.58%, the contracted rate of family doctors is 50.43%, and the demand for contracted services with residents is high in Zhejiang Province [25]. Moreover, another study found that contract behaviour was positively correlated with awareness of family doctors services [23]. Previous studies on CFDS in China focused on the allocation of work resources, significance and difficulties of establishing family doctor system, optimization of contracted service content, exploration of team performance appraisal mechanisms, change of payment mode of medical insurance for contracted services, willingness and satisfaction of contracted services, and work competency and stability [14, 26–29]. Relevant scholars have also proposed some suggestions and directions for the development of CFDS and analysed specific CFDS factors [3, 30].

The ‘Healthy China 2030’ plan was released by the central government in 2016 and is an important long-term national strategic plan. The family doctor service has been regarded as an important tool to achieve Health China 2030 [31] alongside ‘*China improves health services for senior citizens’* (this project aims to prevent and control chronic diseases and promote good health among senior citizens, and calls on family doctors to play a more important role) [32], and *a circular* o*n completing CFDS* (which aims to improve the quality of CFDS and promote the quality and efficiency of CFDS) was published in 2019 [33]. Improving patients/consumers’ willingness to sign contracts with family doctors is a recent policy focus for the government; however, few studies have examined the factors relating to why families sign up to these services and the proportion of policies that include these factors from the perspective of medical staff and patients/consumers, which are worth exploring. Therefore, this study explored the factors associated with CFDS from the perspectives of community health service providers, administrators, medical staff, and patients/consumers, which can help to improve the contracting rate of family doctors, promote the health of consumers, and provide a basis for the government and health administration to formulate policies for CFDS.

## Methods

### Study design and participants

A cross-sectional survey was used to select 12 community health service centres in four regions in Zhejiang, Anhui, Beijing, and Shanghai provinces in a typical area survey. First, a typical city with CFDS in each province was purposively selected. Then, according to the development of CFDS (high, medium, and low), three community health service centres were selected using purposive sampling. The cluster sampling method was used to conduct a questionnaire survey with the directors of the community health service centres, the CFDS team from the community health service agencies, and the administrative staff. Moreover, convenience sampling was used to conduct in-depth interviews with patients/consumers in the same regions. This study mainly combined qualitative (interviews) and quantitative (questionnaire) methods, although only at the methodological level. A questionnaire survey was used to explore the related factors of CFDS from the perspective of medical staff, and interviews were conducted to explore the related factors of CFDS from the perspective of the patients /consumers.

### Data collection

Data were collected from July to September 2017. The respondents completed an anonymous questionnaire after providing informed consent, and a total of 389 questionnaires were distributed. While all of them were returned, 320 questionnaires were valid, and so the total effective rate was 82.3%. The reasons for the elimination of questionnaires included missing data or incomplete questionnaires, multiple omissions, and multiple choices. We interviewed three patients/consumers in each community health service centre. A total of 36 patients/ consumers were interviewed, and the total effective rate was 100.0%.

### Questionnaire

The questionnaire was designed and developed in this study based on the following steps. First, we attempted to select as many items as possible by searching the relevant literature [34, 35]. Next, five experts (including health service management experts, public health experts, and contracted services researchers) were consulted to revise and improve the questionnaire. Finally, a pilot study was conducted at four community health service centres (120 responses were not included in the final data analysis), and the questionnaire was further refined and finalized (Cronbach’s α was 0.865). The final questionnaire comprised the following sections (shown in the Additional file 1):

1. Sociodemographic information (including gender, age, education level, and professional title, etc.).
2. The related factors of CFDS, including 29 items. Using a five-point Likert scale, each item was divided into five levels (very important, important, neutral, unimportant, and very unimportant) according to the influence degree. The participants rated each item according to their own experiences.

### Data analysis

Data were analysed using IBM SPSS Statistics 20.0 and AMOS 21.0, and descriptive statistics were used to analyse the demographic characteristics of participation in community health service agencies. Factor analysis (both exploratory and confirmatory factor analyses) was used to determine the related factors of CFDS [36, 37]. Data is suitable for an exploratory factor analysis if the Kaiser-Meyer-Olkin value is greater than 0.7. The number of factors can be assumed beforehand according to the actual conditions, or the number of dimensions can be determined according to the criterion of eigenvalues greater than 1 or by examining the scree plot. The principal components method was chosen to extract the common factors. Items were excluded according to the following criteria: a factor loading of < 0.40, high loadings on multiple factors, and a factor with less than three items included. Orthogonal rotation was used to determine simple structure, and the method of calculating factor weights was as follows: *ωi* = [(*m* ∑ *j*) *βij* × *ej*]/[(*n* ∑ *i*)(*m* ∑ *j*) *βij* × *ej*]; *Fj* = *β*1*j* × *X*1 + *β*2*j* × *X*2 + *β*3*j* × *X*3 + …… + *βnj* × *Xn*; *Fj* is the principal component (*j* =1, 2, … … m), *X*1, *X*2, *X*3, ……*Xn* for each index, *β*1*j*, *β*2*j*, *β*3*j*, …*βnj* for coefficient score of each index in principal component *Fj*, *ej* is used to express the equation contribution rate of *Fj*. The confirmatory factor analysis model was considered to have a good fit when all of the path coefficients were significant at the level of 0.05; χ2/df was below 5; root mean square error of approximation (RMSEA) was below 0.08; root mean square residual (RMR) was below 0.10; and the goodness of fit index (GFI), normed fit index (NFI), Tucker-Lewis incremental (TLI) fit, and comparative fit index (CFI) were ≥ 0.90. A *p*-value of < 0.05 was considered statistically significant.

## Results

### Results of the in-depth interviews

Through in-depth interviews with patients/consumers, their reasons for being more willing to accept CFDS were identified and summarized. Examples included the patients/consumers’ level of understanding of CFDS; the benefits of CFDS; the concerns about one’s own health; the degree of family doctors’ protection of patients/consumers’ privacy, cost, and process of signing a contract; satisfaction with the community; and advocacy of contracting services.

### Demographic characteristics of community medical staff

Of the 320 respondents, 72.2% were women, 56.6% had received an undergraduate education, and 43.4% had intermediate titles. Among the respondents, general practitioners were the main occupations, and 57.5% of the respondents had working longer than 10 years. The demographic characteristics of the medical personnel are illustrated in Table 1.

**Table 1:** The Demographic characteristics of community medical staff (*N* = 320)

Demographic characteristics	*n*	Percent (%)
Gender		
CMale	89	27.8
Female	231	72.2
Age (years)		
≤ 30	63	19.7
31–40	143	44.7
41–50	90	28.1
>50	24	7.5
Level of education		
<Bachelor	69	21.6
Bachelor	221	69.1
≥ Master	30	9.3
Professional title		
No	14	4.4
Junior	100	31.3
Intermediate	139	43.4
Senior	67	20.9
Professional		
General practitioner	119	37.2
Chinese medicine	25	7.8
Rehabilitation	14	4.4
Nursing	76	23.7
Preventive health care	38	11.9
Administration	24	7.5
Other	24	7.5
Years of experience (years)		
<4	39	12.2
4–7	47	14.7
7–10	50	15.6
>10	184	57.5
Employment form		
Formal employee	228	71.3
Contracted employee	81	25.3
Temporary employee	10	3.1
Other	1	0.3
Monthly income (CNY)		
≤ 2000	16	5.0
2001–4000	63	19.7
4001–6000	124	38.8
6001–8000	85	26.5
≥ 8001	32	10.0

**Analysis of the related factors CFDS.**

### Exploratory factor analysis

The Kaiser-Meyer-Olkin value was 0.836, which demonstrated that the data could be used for the factor analysis (if the Kaiser-Meyer-Olkin value is close to 1, the variable group is suitable for a factor analysis).

After completing the orthogonal rotation of the factor loading matrix, the remaining 25 items had eigenvalues > 1, orthogonal rotation explained the maximum amount of variance, seven factors were extracted from the system, and the cumulative variance contribution rate of 67.613% was shown in the Additional file 2. Table 2 illustrates that 25 observational variables were classified into seven common factors. Based on the results of the group discussion among the project team members, seven factors were named according to the characteristics of the variables observed. F1 was the ‘national policy factor’, the combination of F2 and F3 were the ‘resident factors’, the combination of F4 and F5 were the ‘contract doctor factors’, and the combination of F6 and F7 were the ‘community factors’.

**Table 2:** Rotation component matrix

Items	Component						
	F1	F2	F3	F4	F5	F6	F7
X1 National financial allocations	0.850						
X2 Extent of national policy support	0.838						
X3 Extent of policy support for family doctor service	0.801						
X4 Government propaganda	0.745						
X5 Local government’s investment in special funds for contracted family doctor service	0.708						
X6 Extent of reduction in the incidence of disease because of patients/ consumers signing up for this service		0.850					
X7 Extent of reduction in patients/consumers’ medical costs because of signing up for this service		0.817					
X8 Extent of improvement in the convenience of medical treatment because of signing up for this service		0.736					
X9 Extent to which community patients/consumers trust their family doctors			0.723				
X10 Patients/consumers are satisfied with the contracted services			0.690				
X11 A good medical environment			0.609				
X12 Extent to which patients/ consumers respect, support, and cooperate with family doctors			0.602				
X13 Extent of contracted doctors’ general medical knowledge and mastery of skills				0.857			
X14 Degree of contracted doctors’ health management knowledge and skills				0.775			
X15 Extent of the increase in workload				0.585			
X16 Situation of the first diagnosis of the patients/consumers				0.488			
X17 Awareness of family physician policy					0.790		
X18 Self-working ability					0.726		
X19 Follow the family doctor’s wishes					0.706		
X20 Degree of development of informatization of community medical institutions						0.837	
X21 Completeness of the performance assessment mechanism of the family physician in the community						0.717	
X22 Community medical equipment update and supplement situation						0.711	
X23 Situation that the resident gives the family doctor subsidy after signing a contract							0.814
X24 Incentive mechanism							0.683
X25 Recognition of work by the leadership							0.541

### Confirmatory factor analysis

The results of the confirmatory factor analysis were as follows: the RMSEA was 0.059, and thus was less than the 0.08 cut-off that indicates a good fit; the RMR was 0.05; The TLI, NFI, GFI, and CFI were 0.913, 0.902, 0.905, and 0.917, respectively.

### Results of expert consultations

The health and family planning commission, contracted services researchers, and administrators were selected to carry out an expert consultation. The above results were modified according to their inputs, and the final versions of the predisposing factors are listed below.

In the first round of consultation, the name of each dimension in the model was modified: ‘national policy factors’ were revised to ‘national government factors’, ‘resident factors’ were revised to ‘consumers-related factors’, ‘contracted doctor factors’ was revised to ‘contracted doctor-related factors’, and ‘community factors’ were revised to ‘community health service agency factors’.

The second round of consultation integrated the dimensions of the model. The experts deemed that the ‘situation of the first diagnosis of the patients/consumers’ should be incorporated into the resident-related factors dimension rather than contracted doctor-related factors.

### The final determinants of the CFDS factors

The final determinants of the CFDS factors are shown in Table 3. The related CFDS factors from the perspective of medical staff were divided into four dimensions and 25 items. The four dimensions were named national government factors, community health service agency factors, consumer-related factors, and contracted doctor-related factors, respectively.

**Table 3:** The factors of contracted family doctors services

Dimensions	Component
1. National government factors	1.1 National financial allocations
	1.2 Extent of national policy support
	1.3 Extent of policy support for family doctor service
	1.4 Government propaganda
	1.5Local government’s investment in special funds for contracted family doctor service
2. Community health service agency factors	2.1 Degree of development of informatization of community medical institutions
	2.2 Completeness of the performance assessment mechanism of the family physician in the community
	2.3 Community medical equipment update and supplement situation
	2.4The situation that the resident gives the family doctor subsidy after signing a contract
	2.5 Incentive mechanism
	2.6Recognition of work by the leadership
3. Consumers-related factors	3.1 Extent of reduction in the incidence of disease because of patients/consumers signing up for this service
	3.2 Extent of reduction in patients/consumers’ medical costs because of signing up for this service
	3.3 Extent of improvement in the convenience of medical treatment because of signing up for this service
	3.4 Extent to which community patients/consumers trust their family doctors
	3.5 Patients/consumers are satisfied with the contracted services
	3.6 A good medical environment
	3.7 Extent to which patients/consumers respect, support, and cooperate with family doctors
	3.8 Situation of the first diagnosis of the patients/ consumers
4. Contracted doctor-related factors	4.1 Extent of the increase in workload
	4.2 Mastery knowledge and skills of general practice of contracted doctors
	4.3Mastery knowledge and skills of health management of contracted doctors
	4.4 Awareness of family doctors policy
	4.5 Self-working ability
	4.6 To be the wishes of a family doctor

### Calculation of the factor weights of related CFDS factors from the perspective of medical staff and consumers

The cumulative variance contribution rate of F1–F7 was 67.613% (shown in the Additional file 2). The weighted mean of the variance contribution rate of each factor was calculated, and the evaluation formula of the comprehensive score was obtained: F = (0.302F1 + 0.107F2 + 0.0701F3 + 0.0571F4 + 0.0531F5 + 0.0458F6 + 0.0406F7)/0.676. Indicator weight = composite score/model coefficient.

Based on the above results, we merged similar factors into four dimensions. Finally, the weight coefficients of the common factors were 0.319, 0.247, 0.226, and 0.208, respectively (see Table 4 for specific details).

**Table 4:** Weight and ranking of related factors for family doctor contracted services from the perspective of medical staff

Sequence	Factor	Weight (%)
1	National government factors	31.9
2	Community health service agency factors	24.7
3	Consumers-related factors	22.6
4	Contract doctor- related factors	20.8

Table 5 illustrated that the frequency and ranking of related CFDS factors from the perspective of the consumers. The keywords extracted from the interviews were counted and we found that the frequency sequence of related CFDS factors from the perspective of consumers were as follows: national government factors, contract doctor-related factors, community health service agency factors, and consumers-related factors.

**Table 5:** Frequency and ranking of related factors for family doctor contracted services from the perspective of consumers

Sequence	Factor
1	National government factors
2	Contract doctor- related factors
3	Community health service agency factors
4	Consumers-related factors

## Discussion

Comparing the factors associated with CFDS from the perspectives of patients and of medical staff, we found that the factors of national policy and community health service agency are two important common related factors of CFDS based on the results of interviews, surveys and expert consultations.

The results demonstrated that the national government factor is the main trigger that affected CFDS the most. The government, as the dominant force in the establishment and operation of contracted services, is obligated to ensure the smooth progress of CFDS, to guarantee the fairness and accessibility of services, and conduct the contracted service’s policy.

The government has developed universal health insurance coverage, basic public health service plans, and the national essential drug system, all of which have improved access to and affordability of primary health care [38]. From the perspective of the national government, it is very important to increase financial support for primary health facilities. At the same time, the government should play its part in macroeconomic regulation and control, and combine related departments, such as finance and social security to increase support for inclination to support of community medical institutions, and guide the insured personnel to give priority to primary clinics. In addition, public health services such as health management and health education conducted by family doctors should be included in the scope of medical insurance. CFDS should be linked with medical insurance, facilitating family doctors’ gatekeeper role in terms of medical insurance control fees and health management.

The results demonstrated that the community health service institutions factor is also one of the related CFDS factors. The community health service agency is the executor of the contracted service’s policy, and the extent of its power of execution directly determines the direction of CFDS, and the completeness of the hardware and software facilities of a community health service organization is the basis of its power of execution. Therefore, the degree of development of its informatization, medical facilities, and equipment within the institution, and performance assessment and incentive mechanisms are all necessary conditions for the promotion of contracted services. At the same time, the supporting hardware facilities of the community health service institutions, including equipment and drugs are insufficient, which impedes the development of CFDS. However, family doctor team members, as important stakeholders of the medical alliance, can mobilize their enthusiasm and achieve the sustainable development of contracted services only if they are allowed to obtain reasonable benefits from contracted service operations [39]. Therefore, some suggestions are necessary from the perspective of community health institutions. First, community health institutions should speed up the development of a unified information platform to achieve dynamic management of contracted patients’/consumers’ information and the analysis of the dynamics of consumers through real-time data monitoring. At the same time, community health agencies should increase investment in hardware facilities, thereby making medical services more accessible to patients/consumers, changing their views on primary medical care and increasing satisfaction with primary health services [38]. The most important point is that salary not only meets the basic survival needs of employees, but also is a kind of recognizing and respecting employee performance and contributions [40]. Therefore, community health agencies should establish a sound supporting performance appraisal system and incentive mechanism, to increase employees’ enthusiasm toward their work, helping them better serve patients/consumers.

Consumer-related factors have a great impact on contracted services. As important stakeholders involved in the reform of China’s health care and CFDS, patients/consumers are mainly concerned with medical technology, medical expenses, emotional support, and respect. With the development of the medical system and the improvements in its economic development, patients/consumers’ demands for overall medical services have been increasingly met, and service quality is a key factor in attracting patients/consumers. The results of the survey and interviews suggest that patients/consumers are not confident about the qualifications and medical technology of family doctors and community health agencies. The government must create an atmosphere of public opinion that understands and supports the services of family doctors, and reasonable contracting fees need to be formulated. We also recommend that residents respect, support, and cooperate with family doctors.

Factors related to contracted doctors also influence CFDS. Family doctors and team members are the flag-bearers of CFDS, and their service capabilities, willingness, and attitudes all influence its smooth development. According to the ‘Guiding Opinions of the State Council on Establishing the General Practitioner System’, training qualified family doctors by vigorously carrying out transfer training for community doctors and raising the academic qualification level of community doctors can also reduce the workload of existing family doctors and teams, thus providing more effective health care. Moreover, community health organizations should strengthen the talent team construction of family doctors in all aspects, conduct regular training for family doctor teams, and attract graduates of general medicine or specialists in second and third-level hospitals who have undergone standardized training. In addition, the service quality of community health service centres and the level of medical technology should be improved. This will increase patients/consumers’ sense of identity and belonging, and improve their satisfaction. At the time of diagnosis, the team of family doctors should pay attention to service attitude, reduce the distance between themselves and patients, and provide humanistic care.

### Limitations

This study has two limitations. First, the sample size of the study was small; a typical survey needs to select a sample representative of a typical unit, so the researchers need to exercise particularly good judgement, or their conclusions may be biased, and it may be difficult to generalise from the results of a typical survey to the overall situation. Second, differences in self-perception between medical staff and patients may have biased their responses, affecting the outcomes. Future research can further improve the overall design and reduce the impact of cognitive differences on outcomes. Third, this study only refines several related factors but did not indicate the mechanism of which factor worked.

## Conclusions

National governments, community health agencies, community health workers, and consumers play an important role in the advancement of CFDS. Therefore, the development of CFDS needs to consider the rights and interests of all stakeholders involved.

## Supplementary information

**Additional file 1: Questionnaire S1.** Influencing factors of contracted services of family doctors.

**Additional file 2: Table S1.** Explained total variance.

## Data Availability

The datasets used and/or analysed during the current study are available from the corresponding author on reasonable request.

## Abbreviations

CFDS: Contracted Family Doctors Services
CFI: Comparative fit index
GFI: Goodness of fit index
NFI: Normed fit index
RMR: Root mean square residual
RMSEA: Root mean square error of approximation
TLI: Tucker-Lewis incremental fit
