# Supplementary material for: Factors associated with contracted services of Chinese family doctors from the perspective of medical staff and consumers: a cross-sectional study
Source: BMC Health Serv Res. 2019 Dec 21;19:986. doi: 10.1186/s12913-019-4801-y (PMC6925852; doi:10.1186/s12913-019-4801-y)
Supplement: Supplementary file 1 — Additional file 1: Questionnaire S1. Influencing factors of contracted services of family doctors. [file 12913_2019_4801_MOESM1_ESM.docx]

**Questionnaire on influencing factors of contracted services of family doctors**

**Demographic characteristics**

1. Gender：（1）Male （2）Femalek

2. Age: （1）≤30 （2）31-40 （3）41-50 （4）＞50

3. Level of education：（1）＜Bachelor（2）Bachelor（3）≥Master

4. Professional title：（1）No（2）Junior（3）Intermediate（4）Senior

5.Professional：（1）General practitioner（2）Chinese medicine（3）Rehabilitation（4）Nursing（5）Preventive health care（5）Administration（6）Other

6. Years of experience (years)：（1）＜4（2）4-7（3）7-10（4）＞10

7. Employment form：（1）Formal employee（2）Contracted employee（3）Temporary employee（4）Other

8. Monthly income (CNY)： （1）≤2000元 （2）2001—4000元 （3）4001—6000 元

（4）6001—8000元（5）≥8001

**Influencing factors of contracted services of Chinese family doctors**

| **Please tick "√" in the box of corresponding score** | **very important** | **important** | **neutral** | **unimportant** | **very unimportant** |
| --- | --- | --- | --- | --- | --- |
| 1. Extent of national policy support | **5** | **4** | **3** | **2** | **1** |
| 2. National financial allocations | **5** | **4** | **3** | **2** | **1** |
| 3. Social recognition | **5** | **4** | **3** | **2** | **1** |
| 4. Extent of policy support for family doctor service | **5** | **4** | **3** | **2** | **1** |
| 5. Local government’s investment in special funds for contracted family doctor service | **5** | **4** | **3** | **2** | **1** |
| 6. Technical support | **5** | **4** | **3** | **2** | **1** |
| 7. Publicity degree of community to contracted services for family doctors | **5** | **4** | **3** | **2** | **1** |
| 8. Completeness of the performance assessment mechanism of the family physician in the community | **5** | **4** | **3** | **2** | **1** |
| 9. Degree of development of informatization of community medical institutions | **5** | **4** | **3** | **2** | **1** |
| 10. Community medical equipment update and supplement situation | **5** | **4** | **3** | **2** | **1** |
| 11. Whether the community is a pilot unit of contracted services for family doctors | **5** | **4** | **3** | **2** | **1** |
| 12. Incentive mechanism | **5** | **4** | **3** | **2** | **1** |
| 13. Situation that the resident gives the family doctor subsidy after signing a contract | **5** | **4** | **3** | **2** | **1** |
| 14. Reasonable income | **5** | **4** | **3** | **2** | **1** |
| 15. Recognition of work by the leadership | **5** | **4** | **3** | **2** | **1** |
| 16. Extent of reduction in the incidence of disease because of patients/ consumers signing up for this service | **5** | **4** | **3** | **2** | **1** |
| 17. Extent of reduction in patients/consumers’ medical costs because of signing up for this service | **5** | **4** | **3** | **2** | **1** |
| 18. Extent of improvement in the convenience of medical treatment because of signing up for this service | **5** | **4** | **3** | **2** | **1** |
| 19. A good medical environment | **5** | **4** | **3** | **2** | **1** |
| 20. Patients/consumers are satisfied with the contracted services | **5** | **4** | **3** | **2** | **1** |
| 21. Extent to which community patients/consumers trust their family doctors | **5** | **4** | **3** | **2** | **1** |
| 22. Extent to which patients/ consumers respect, support, and cooperate with family doctors | **5** | **4** | **3** | **2** | **1** |
| 23. Situation of the first diagnosis of the patients/consumers | **5** | **4** | **3** | **2** | **1** |
| 24. Extent of the increase in workload | **5** | **4** | **3** | **2** | **1** |
| 25. Extent of contracted doctors’ general medical knowledge and mastery of skills | **5** | **4** | **3** | **2** | **1** |
| 26. Degree of contracted doctors’ health management knowledge and skills | **5** | **4** | **3** | **2** | **1** |
| 27. Self-working ability | **5** | **4** | **3** | **2** | **1** |
| 28. Awareness of family physician policy | **5** | **4** | **3** | **2** | **1** |
| 29. Follow the family doctor’s wishes | **5** | **4** | **3** | **2** | **1** |
