# Supplementary material for: Factors associated with contracted services of Chinese family doctors from the perspective of medical staff and consumers: a cross-sectional study
Source: BMC Health Serv Res. 2019 Dec 21;19:986. doi: 10.1186/s12913-019-4801-y (PMC6925852; doi:10.1186/s12913-019-4801-y)
Supplement: Supplementary file 2 — Additional file 2: Table S1. Explained total variance. [file 12913_2019_4801_MOESM2_ESM.docx]

**Table S1. Explained total variance**

| Components | Initial Eigenvalues | | | Extract Variance Sum Loading | | | Rotating Variance Sum Loading | | |
| --- | --- | --- | --- | --- | --- | --- | --- | --- | --- |
|  | total | variance (%) | cumulative (%) | total | variance (%) | cumulative (%) | total | variance (%) | cumulative (%) |
| 1 | 7.549 | 30.196 | 30.196 | 7.549 | 30.196 | 30.196 | 3.483 | 13.932 | 13.932 |
| 2 | 2.685 | 10.742 | 40.938 | 2.685 | 10.742 | 40.938 | 2.600 | 10.401 | 24.333 |
| 3 | 1.754 | 7.016 | 47.954 | 1.754 | 7.016 | 47.954 | 2.370 | 9.480 | 33.813 |
| 4 | 1.428 | 5.713 | 53.667 | 1.428 | 5.713 | 53.667 | 2.280 | 9.121 | 42.935 |
| 5 | 1.328 | 5.314 | 58.981 | 1.328 | 5.314 | 58.981 | 2.151 | 8.605 | 51.540 |
| 6 | 1.143 | 4.570 | 63.551 | 1.143 | 4.570 | 63.551 | 2.136 | 8.546 | 60.086 |
| 7 | 1.015 | 4.062 | 67.613 | 1.015 | 4.062 | 67.613 | 1.882 | 7.527 | 67.613 |
| 8 | 0.890 | 3.560 | 71.172 |  |  |  |  |  |  |
| 9 | 0.862 | 3.446 | 74.619 |  |  |  |  |  |  |
| 10 | 0.795 | 3.181 | 77.800 |  |  |  |  |  |  |
| 11 | 0.699 | 2.797 | 80.597 |  |  |  |  |  |  |
| 12 | 0.601 | 2.404 | 83.001 |  |  |  |  |  |  |
| 13 | 0.513 | 2.053 | 85.054 |  |  |  |  |  |  |
| 14 | 0.480 | 1.921 | 86.975 |  |  |  |  |  |  |
| 15 | 0.432 | 1.730 | 88.705 |  |  |  |  |  |  |
| 16 | 0.426 | 1.705 | 90.410 |  |  |  |  |  |  |
| 17 | 0.408 | 1.634 | 92.044 |  |  |  |  |  |  |
| 18 | 0.363 | 1.451 | 93.494 |  |  |  |  |  |  |
| 19 | 0.317 | 1.269 | 94.764 |  |  |  |  |  |  |
| 20 | 0.288 | 1.151 | 95.914 |  |  |  |  |  |  |
| 21 | 0.273 | 1.093 | 97.008 |  |  |  |  |  |  |
| 22 | 0.222 | 0.888 | 97.895 |  |  |  |  |  |  |
| 23 | 0.209 | 0.834 | 98.730 |  |  |  |  |  |  |
| 24 | 0.176 | 0.705 | 99.434 |  |  |  |  |  |  |
| 25 | 0.141 | 0.566 | 100.000 |  |  |  |  |  |  |
